# Capacitively-Coupled ECG and Respiration for Sleep–Wake Prediction and Risk Detection in Sleep Apnea Patients

**DOI:** 10.3390/s21196409

**Published:** 2021-09-25

**Authors:** Dorien Huysmans, Ivan Castro, Pascal Borzée, Aakash Patel, Tom Torfs, Bertien Buyse, Dries Testelmans, Sabine Van Huffel, Carolina Varon

**Affiliations:** 1 STADIUS Center for Dynamical Systems, Signal Processing and Data Analytics, Department of Electrical Engineering (ESAT), KU Leuven, 3001 Leuven, Belgium; sabine.vanhuffel@esat.kuleuven.be (S.V.H.); carolina.varon@esat.kuleuven.be (C.V.); 2 Circuits and Systems for Health, Imec-Leuven, 3001 Leuven, Belgium; ivand.castro@imec.be (I.C.); Aakash.Patel@imec.be (A.P.); tom.torfs@imec.be (T.T.); 3 Department of Pneumology, UZ Leuven, 3000 Leuven, Belgium; pascal.borzee@uzleuven.be (P.B.); bertien.buyse@uzleuven.be (B.B.); dries.testelmans@uzleuven.be (D.T.); 4 Service de Chimie-Physique E.P., Université Libre de Bruxelles, 1050 Brussels, Belgium

**Keywords:** sleep apnea, signal quality, capacitively-coupled bioimpedance, sleep–wake prediction

## Abstract

Obstructive sleep apnea (OSA) patients would strongly benefit from comfortable home diagnosis, during which detection of wakefulness is essential. Therefore, capacitively-coupled electrocardiogram (ccECG) and bioimpedance (ccBioZ) sensors were used to record the sleep of suspected OSA patients, in parallel with polysomnography (PSG). The three objectives were quality assessment of the unobtrusive signals during sleep, prediction of sleep–wake using ccECG and ccBioZ, and detection of high-risk OSA patients. First, signal quality indicators (SQIs) determined the data coverage of ccECG and ccBioZ. Then, a multimodal convolutional neural network (CNN) for sleep–wake prediction was tested on these preprocessed ccECG and ccBioZ data. Finally, two indices derived from this prediction detected patients at risk. The data included 187 PSG recordings of suspected OSA patients, 36 (dataset “Test”) of which were recorded simultaneously with PSG, ccECG, and ccBioZ. As a result, two improvements were made compared to prior studies. First, the ccBioZ signal coverage increased significantly due to adaptation of the acquisition system. Secondly, the utility of the sleep–wake classifier increased as it became a unimodal network only requiring respiratory input. This was achieved by using data augmentation during training. Sleep–wake prediction on “Test” using PSG respiration resulted in a Cohen’s kappa (κ) of 0.39 and using ccBioZ in κ = 0.23. The OSA risk model identified severe OSA patients with a κ of 0.61 for PSG respiration and κ of 0.39 using ccBioZ (accuracy of 80.6% and 69.4%, respectively). This study is one of the first to perform sleep–wake staging on capacitively-coupled respiratory signals in suspected OSA patients and to detect high risk OSA patients based on ccBioZ. The technology and the proposed framework could be applied in multi-night follow-up of OSA patients.

## 1. Introduction

Obstructive sleep apnea (OSA) is a sleep disorder with a high prevalence among the general population, but remains heavily underdiagnosed [1,2]. It is characterized by breathing disturbances during sleep, causing hypoxemia, large chest motions, and arousals from sleep, which fragment the patient’s sleep and reduce phases of rapid eye movement (REM) and slow wave sleep. Consequently, OSA is a recognized risk factor for excessive daytime sleepiness, hypertension, and cardiovascular diseases [3]. Its severity is measured by the Apnea–Hypopnea Index (AHI), i.e., the number of respiratory events per hour of sleep. In order to determine the AHI and diagnose sleep apnea, an overnight sleep study is required. A patient is then categorized as not suffering from OSA if 0⩽ AHI <5, as mild OSA if 5⩽ AHI <15 with presence of symptoms, moderate OSA if 15⩽ AHI <30, or severe OSA if AHI ⩾ 30 [4]. The procedure is, however, expensive, as well-trained staff and a multitude of sensors are required. These sensors include, among others, electrocardiography (ECG), respiratory inductance plethysmography (RIP), oxygen saturation, electroencephalogram, electrooculogram, and electromyogram. As such, the extensive setup also poses a high level of discomfort for the patient, which inhibits a normal sleeping pattern.

To enable screening within the general population and a long-term follow up of patients, home-monitoring systems are required. Many emerging unobtrusive sensor technologies for sleep-related monitoring are based on cardiac and respiratory signals. Firstly, these signals carry information about the different sleep stages and occurring apneas, in the form of patterns of bradycardia, tachycardia and respiratory disturbances. Secondly, these can be acquired more comfortably as opposed to the electroencephalogram and oxygen saturation. Huysmans et al. developed a sleep–wake classification network for OSA patients based on convolutional neural networks (CNN) [5]. In addition, the predicted hypnogram served as input for an OSA patient detection model. Both the network and model were exclusively trained on ECG and RIP data from PSG. Nevertheless, these were specifically designed to process single short-term segments, as unobtrusive data is likely to be affected by artefacts and data loss. For long-term monitoring, capacitively-coupled ECG (ccECG) and capacitively-coupled bioimpedance (ccBioZ) are likely to be suitable modalities as these sensors do not require a direct contact to the skin. Instead, the skin and electrode serve as electric conductors with any clothing and fabric as the dielectric medium, to generate a capacitive coupling. In this study, the ccECG and ccBioZ sensors integrated in a mattress, previously presented in [6], were used. Few sleep monitoring studies have been performed with these sensor types. Lee et al. tested the potential of their ccECG recording system for detection of REM sleep and wakefulness through heart rate variability parameters [7]. Furthermore, Kido et al. classified the sleeping position from ccECG as a step towards personal healthcare [8]. Deviaene et al. applied the ccECG and ccBioZ on an apnea detection algorithm [9].

This study has three objectives, of which the first one is related to the technology evaluation and the remaining two to the clinical application. The technology objective is to assess the quality of the ccECG and ccBioZ on a larger patient database compared to the one presented in [10]. Specifically, there is a focus to further understand the performance of the ccBioZ signal by applying recently developed signal quality indicators (SQIs) [11] and performing a comparison against the respiratory PSG signal. The first application objective is to further develop the sleep–wake classifier of [5] to be used with ccECG and ccBioZ. The original classifier is first applied on the new data and finetuned afterwards. The final classification outcome based on the capacitively-coupled data is used to predict the patient’s sleep architecture or “hypnogram”. However, it is hypothesized that the restlessness and movements induced by apneas of OSA patients will heavily affect the unobtrusive recordings and deteriorate the performances of sleep–wake classification. To the best of our knowledge, this is the first study to investigate automated sleep–wake prediction in suspected OSA patients based on ccECG and ccBioZ. The second application objective is to exploit the unobtrusively predicted hypnogram to detect patients with an increased risk of suffering from OSA. In this way, patients can be prioritized directly for a clinical diagnostic test. Hence, this study addresses the need for unobtrusive monitoring of suspected OSA patients.

## 2. Materials

The dataset consisted of 187 patients with suspected sleep apnea. They underwent a diagnostic PSG at the sleep laboratory of the University Hospitals Leuven (UZ Leuven, Belgium). The ethical committee of UZ Leuven approved the data collection (S60319) and all patients signed an informed consent form. The B3IP device from Medatec (Haillot, Belgium) served as polysomnograph and provided data from the built-in ECG (SPES electrodes) and built-in thoracic RIP (SleepSense belts). The Medatec Brainnet Winacq 5.0 and Medatec Brainnet Winrel 5.0 were the acquisition and the analyzing software, respectively. The ECG and RIP were sampled at 500 Hz. Based on the PSG data, a clinical sleep expert annotated the sleep stages and apneas according to the AASM 2012 scoring rules [12].

The complete patient dataset was split into subsets for network optimization and independent model testing. Training of the sleep–wake classifier was performed on 39 patients (CNN_Train), with validation on 17 patients (CNN_Val) and testing on 26 patients (CNN_Test). These datasets only included patients with AHI < 10, and the use of these datasets is further explained in Section 3.3. Next, dataset CNN_Test was merged with patients with higher AHI and split again according to OSA severity in the subsets No (AHI < 5), Mild (5 ⩽ AHI < 15), Mod (15 ⩽ AHI < 30), and Sev (AHI ⩾ 30). These datasets were applied for testing the sleep–wake classifier and model optimization for OSA patient detection.

**Figure 1:**
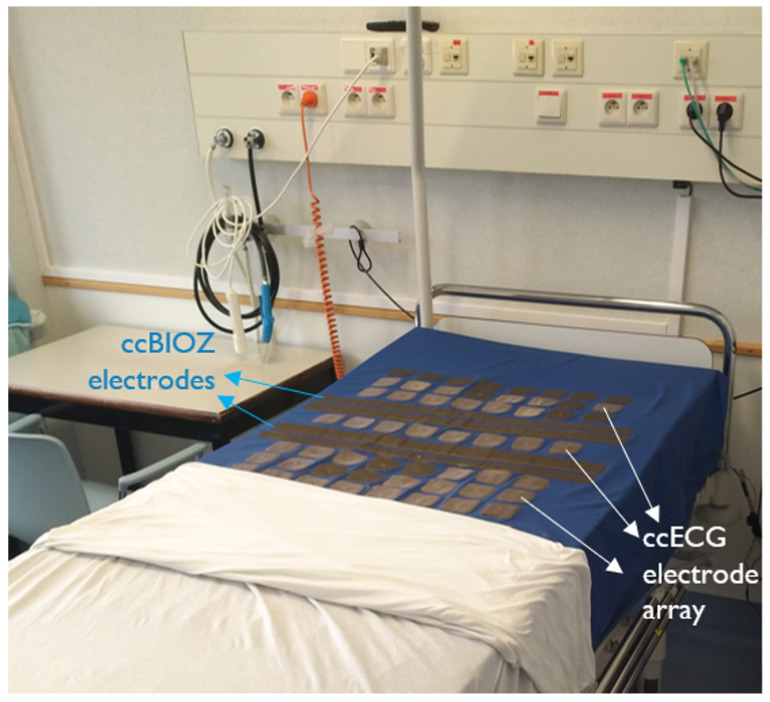
Mattress with integrated capacitively-coupled sensors for acquisition of ccECG and ccBioZ.

Out of 187 patients, 40 patients were part of an additional data collection, complying with the same ethical standards. Their PSG recordings were acquired simultaneously with ccECG and ccBioZ sensors embedded in a mattress, with the setup illustrated in Figure 1. The ccECG and ccBioZ were acquired as described in [6], at a sampling frequency of 512 Hz. Due to technical or handling problems (e.g., power cable disconnection), data was correctly collected on 36 patients. This data was then used both to perform a technology evaluation in combination with signal quality indicator (SQI) algorithms, as well as to perform an independent validation of the sleep–wake classification and detection of OSA patients. Because of this, these 36 patients were left out as unseen dataset test. The four PSG recordings of patients with missing capacitively-coupled data were ignored to allow statistical tests with repeated measurements within the Test dataset. As such, dataset Test contained 6 non-apneic patients, 4 mild, 9 moderate, and 17 severe OSA patients. The complete overview of datasets can be found in Table 1.

## 3. Methods

**Figure 2:**
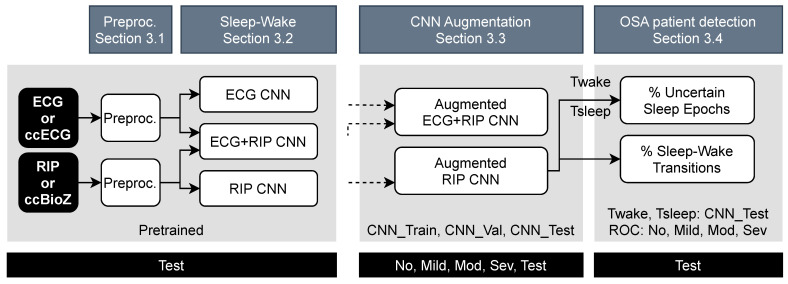
Pipeline of the study. The top blocks refer to the sections elaborating on the procedure below. The grey background blocks indicate the dataset used for model training and optimization. The black blocks at the bottom define the used test data. The data were first evaluated against PSG gold standard, then preprocessed and applied onto the original sleep–wake classifiers. Then, the RIP network was improved and merged with the ECG network. The augmented RIP CNN resulted in sleep–wake predictions, from which the percentage of uncertain predicted sleep epochs was derived, as well as the percentage of sleep stage transitions. These two indices were combined for OSA patient detection.

The PSG and capacitively-coupled signals were first preprocessed by synchronization between the modalities and determination of high-quality data segments. ccECG and ccBioZ data were then evaluated in terms of SQI-based coverage and the agreement of extracted features (e.g., beat-to-beat heart rate, respiration rate) with the gold standard PSG signal. Following this, the data were normalized prior to feeding the CNN (Section 3.1). Then, the performance of the original sleep–wake classifier was tested on the unobtrusive data (Section 3.2). This was followed by a classifier improvement step using data augmentation (Section 3.3). Finally, patients at risk of OSA were detected using indices derived from their predicted sleep–wake pattern. This prediction was based on their capacitively-coupled recordings (Section 3.4). The full pipeline of the study is illustrated in Figure 2.

### 3.1. Data Preprocessing

#### 3.1.1. Quality Assessment and Technology Evaluation against Gold Standard

After acquisition of the raw ccECG and ccBioZ, the signals were processed to provide signals of the highest possible quality. This phase was mainly based on the work of Castro et al. in [13,14] for ccECG signals and of Albaba et al. [11] for ccBioZ signals.

**ccECG:** The ccECG was acquired by a multi-channel system, producing four simultaneous ccECG channels [6]. These were all synchronized with the reference ECG and subsequently split into 30 s segments (or “epochs”). For every epoch, the highest quality channel was selected. In this way, a single aggregated ccECG signal was provided [13]. Next, every segment of the aggregated ccECG was either labeled as high or low quality using a high-threshold SVM model [14]. Technology evaluation for the ccECG signals comprised determining the SQI-based coverage as the percentage of high-quality data, followed by a feature comparison against the information extracted from the PSG ECG. This included beat detection sensitivity, tachogram correlation, and R-R interval errors, with R-R the time interval between two consecutive beats. In addition, coverage metrics were compared against a previous dataset described in [10].

**ccBioZ:** The ccBioZ was a single channel signal [6], synchronized to the reference RIP and segmented using a 30 s window. The technology evaluation of the ccBioZ signals aimed to evaluate an improved electronic circuit for acquisition of the respiratory activity. ccBioZ signals were therefore evaluated in terms of their SQI-based coverage. The same approach was taken as for ccECG, but with application of the recently developed ccBioZ SQI algorithms (presented in [11]). Furthermore, both the SQI-preprocessed data as well as the complete dataset was compared against the gold standard PSG RIP signal. This included metrics of respiration rate error (both in frequency and time domain) as well as the average correlation per patient of the ccBioZ signal with the PSG RIP signal. Finally, this was compared against the data in [10] applying the same processing. It is noted that the current study included more patients than prior data collection in [10].

#### 3.1.2. Sleep–Wake Classification

The subsequent preprocessing was specifically designed for sleep–wake classification using a dedicated CNN classifier proposed in [5].

**(cc)ECG:** Heart beats were detected in 30 s (cc)ECG segments, using the method proposed in [15]. Segments with less than 15 detected beats were removed. In each remaining segment, the first beat was slightly adjusted in time by linear extrapolation, as there could be a relatively wide time gap between the segment start and the first detected beat. This possibly causes border problems during interpolation in a later step. Thus, the first beat time was defined as the second beat time minus the mean of the second and third beat interval. The same border effect could occur at the last beat as well. Thus, its time was redefined as the second last beat time added with the mean of the second and third last beat interval. Next, the heart rate (HR) at every beat was extracted. The HR was calculated in beats per minute using HR = 60/R-R. The HR values were subsequently interpolated at 4 Hz to generate a tachogram. Next, HR outliers were identified whenever the HR was outside the range of 40 to 180 BPM, outside the segment’s median value ± 20 BPM, or outside the segment’s median value ± (3 × the segment’s standard deviation (SD)). Next, the outliers were corrected as long as the duration was smaller or equal to 2.5 s. Segments with outliers of longer duration were removed. This outlier gap was filled by mirroring the values preceding the gap [16]. This was followed by subtracting the signal with the patient’s overall median BPM value.

**BioZ/RIP:** The 30 s BioZ or RIP segments were bandpass filtered using a Butterworth filter with cutoff frequencies at 0.04 Hz and 2 Hz. Segments were then downsampled to 4 Hz. Every segment was then normalized by subtraction of the patient’s overall median value and division by the patient’s overall SD. Next, the individual median per segment was subtracted.

### 3.2. CNN-Based Sleep–Wake Classification

**Figure 3:**
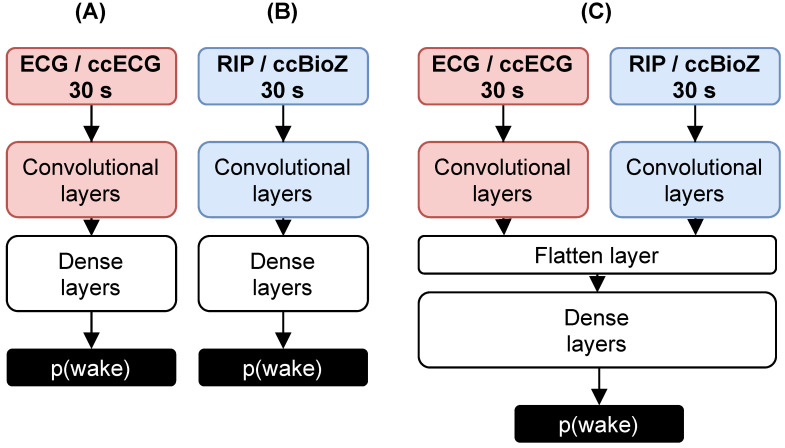
Sleep–wake classifiers. (**A**) Unimodal network for cardiac input and (**B**) for respiratory input. (**C**) Multimodal network consisting of two branches. The left branch received tachograms and the right branch received respiratory waveforms.

The multimodal sleep–wake network proposed in [5] was based on a CNN consisting of two branches, illustrated in Figure 3. One branch received the 30 s tachograms and the other branch the filtered 30 s respiratory effort segments. Every modality could also serve separately as an input to the corresponding unimodal CNN. Training of these networks was performed on PSG recordings. To verify the generalization capability of the unimodal networks on the novel capacitively-coupled modalities, the ccECG was fed to the original cardiac network and the ccBioZ to the respiratory network. For this, dataset Test was applied, which included 36 capacitively-coupled recordings (see Table 1). Only the high-quality segments were taken into account, as an accurate heart beat detection was important for the ccECG and to have clean waveforms for the ccBioZ. It was expected that the cardiac network generalizes well, as the tachogram of the ccECG should be similar to the reference [13]. The respiratory network, on the other hand, was based on waveforms. This potentially causes a loss in performance, as different wave characteristics and different amplitude changes can be expected in the ccBioZ signal.

### 3.3. RIP Network Augmentation

Since a lower generalization of the respiratory model was expected, normalization of the respiratory signal was redefined and the RIP network was trained with data augmentation. Finally, the augmented RIP network was merged with the ECG network to create a multimodal network and to investigate performance differences.

#### 3.3.1. Input Normalization

The normalization of the RIP signal during preprocessing (see Section 3.1.2) ensured that the signal had a standard deviation of one, with a median around zero. This ensured the stability of the CNN learning process and a similar influence of all data segments. However, it does not prevent the network from learning sleep–wake patterns based on the amplitude of the input segment. For example, during deep sleep, the patient’s breathing is deeper and generates an RIP signal with larger amplitude. However, the relative change relies heavily on the type of sensor used. In addition, if the ccBioZ data contained many artefacts, the overall SD for normalization could become very large. Normalizing the signal by division with the SD would then result in a small signal amplitude. Therefore, a *min-max* normalization scheme could be chosen, with scaling of *every* segment between 0 and 1. This garantueed that the ccBioZ segments had a similar amplitude range to the RIP segments. As a zero median is preferred for CNN training, a scaling between −0.5 and 0.5 was performed.

#### 3.3.2. Data Augmentation

To further reduce the amplitude dependency and to increase the training set size, the CNN was trained with data augmentation. First, the recording of every patient was duplicated four times. Next, with every training step, each 30 s data segment was scaled with a random factor between 0.2 and 3.2. These ranges were chosen to keep the amplitude between reasonable ranges. This procedure should increase the number of learning examples and reduce the dependency of the network to changes in amplitude.

#### 3.3.3. RIP Network Retraining

The RIP network was retrained with this data augmentation procedure and using the PSG recordings from patients with relatively low AHI (AHI < 10, as defined in [5]). This forced the network to learn patterns related to sleep stages, and not those related to apneas. CNN_Train contained 70% (N=39) of the data subset for CNN weight training and CNN_Val contained 30% (N=17) for validation, with *N* being the number of patients. The splitting of the dataset was repeated ten times, using a different seed for randomization, to train ten RIP networks. The network with the highest Cohen’s Kappa score (κ) on a separate test set (CNN_Test) was selected. This dataset only included patients with an AHI < 10, but the selected network was also tested on patients with higher AHI. For this, CNN_Test was merged with the remaining 65 recordings and split again according to the conventional OSA classes into datasets No, Mild, Mod, and Sev (see Table 1).

#### 3.3.4. Multimodal Network

The selected augmented RIP network was merged with the ECG network to evaluate the contribution of the latter to the performance of the RIP network. First, the ECG network was trained using the same split for CNN_Train and CNN_Val. Then, the trained convolutional layers of the RIP and ECG network were combined by dense layers (see Figure 3). The weights of the convolutional layers were frozen, and the dense layers were retrained by CNN_Train and CNN_Val. The same workflow as described in [5] was used. A Wilcoxon signed rank test verified the performance differences between classifiers or datasets, which is a non-parametric test for repeated measurements.

### 3.4. Detection of OSA Patients

In [5], it was shown that sleep–wake predictions become uncertain in the presence of apneas as the network is trained on patients with relatively few apneas. In addition, apneas disturb the characteristic sleep patterns affecting both the cardiac and respiratory signals. As a result, the percentage of uncertain sleep predictions of the classifier is related to the severity of OSA. Thus, a discrimination was made between *confident* and *uncertain* predicted epochs. The probability of wakefulness p(wake) for a wake-predicted epoch should reach the threshold $T_{wake}$ to be labeled confident. $T_{wake}$ was defined by median p(wake) of epochs predicted as wake minus its SD, calculated over the patients in CNN_Test. Similarly, p(sleep) of confident sleep predicted epochs should exceed $T_{sleep}$, which was the median p(sleep) of epochs predicted as sleep minus its SD. After defining these thresholds based on the predicted PSG recordings of CNN_Test, the percentage of uncertain sleep epochs in the ccBioZ was calculated. This was the first index for OSA patient detection as proposed in [5]. A second index was the percentage of sleep–wake and wake–sleep transitions, as there was an expected increase in sleep fragmentation, sympathetic activation, and micro-awakenings.

For simplicity and similarly to previous study ([5]), it was chosen that if *at least one of both metrics* exceeded a selected threshold, the patient was identified as being at risk of OSA (AHI ⩾ 15), i.e., detected positive. These thresholds were selected by means of an ROC analysis, as the values that correspond to a high specificity for OSA detection. The goal was to detect moderate and severe OSA patients at home using the unobtrusive sensors, so they could be prioritized for a diagnostic PSG.

The ccBioZ can be very sensitive to movement due to its unobtrusive nature. This can reduce the signal quality in epochs with increased movement. This means that more epochs with a lower quality can be expected for OSA patients, due to their general restlessness and apneas that evoke large body movements when breathing is restored. In addition, apneas disrupt the regularity of the respiratory signal due to breathing cessations and the cardiac signal due to tachycardia and bradycardia. Thus, the effect of apneas causing (false) wake predictions is expected to be enhanced by respiration monitoring with a capacitively-coupled device.

As a benefit, apnea segments were hypothesized to be informative for detection of patients with high OSA risk. However, when using only high-quality ccBioZ segments, it is expected that only apneas with more subtle physiological reactions are retained. This idea is supported by Deviaene et al. in [9], who observed a decreased sensitivity for apnea detection when using only high-quality capacitive data. Thus, the procedure for OSA patient detection was assessed with the full ccBioZ recordings (i.e., including both high and low quality segments). The performance was compared to the high-quality ccBioZ dataset by means of the Cohen’s Kappa score (κ), accuracy (Acc), sensitivity (Se), specificity (Sp), and diagnostic odds ratio (DOR). The DOR is a single metric of performance, independent of the prevalence and is defined as DOR $=\frac{Se\times Sp}{\left(1-Se)\times (1-Sp\right)}$.

## 4. Results

### 4.1. Data Quality Assessment and Technology Evaluation against Gold Standard

**Figure 4:**
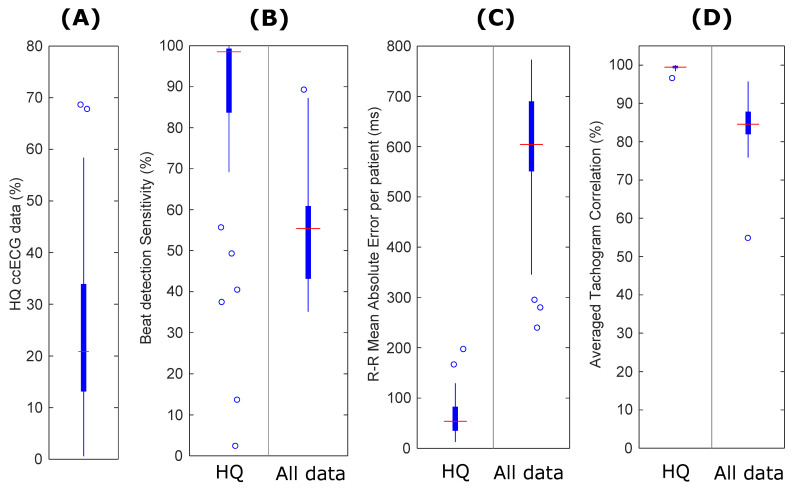
Results of ccECG evaluation when applying SQIs (high-quality data, HQ) and comparing against gold standard PSG ECG. The SQI algorithms are able to extract 21% of high-quality data for feature extraction. This is lower than in the previous data collection [10], presumably due to degradation of the ECG electrodes over time by usage and cleaning of the mattress. (**A**) Percentage of data used per patient after SQI processing. (**B**) Beat detection sensitivity before and after applying SQIs. (**C**) R-R Mean Absolute Error before and after applying SQIs. (**D**) Averaged tachogram correlation with gold standard before and after applying SQIs.

The ccECG evaluation results when applying the SQIs and comparing against gold standard PSG ECG are shown in Figure 4. Figure 4A shows the distribution of the per-patient percentage of ccECG data considered of high quality when applying SQI algorithms, with a median of 20.8% and a 25–75 percentile in the range of 13.1–33.1%. This shows a reduction in the amount of usable data with respect to a previous data collection, which had a median of 37.1% coverage when evaluating 11 OSA patients [10]. Nevertheless, it is clear that the SQI algorithms are able to automatically identify the high-quality data, as comparison of the high-quality data against PSG ECG results in a high performance, shown in Figure 4B–D.

**Figure 5:**
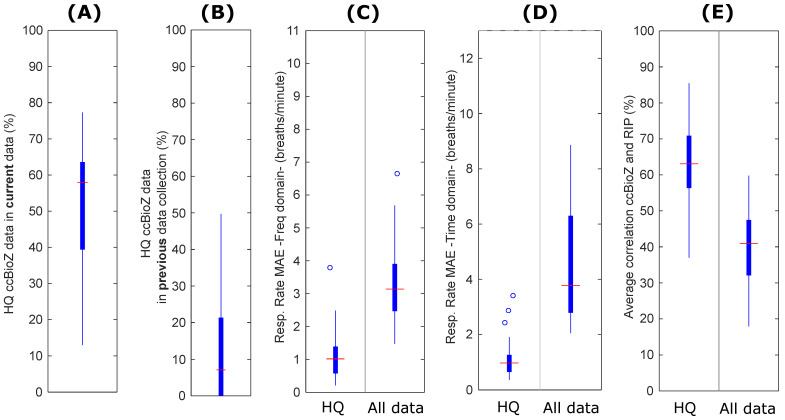
Results of ccBioZ evaluation when applying SQIs (high-quality data, HQ) and comparing against gold standard PSG RIP. The increase in (**A**) percentage of ccBioZ classified as HQ in the current study is clear, when comparing to (**B**) the HQ ccBioZ data from a previous data collection [10]. (**C**,**D**) show the respiration rate error with and without SQI processing. (**E**) shows the average correlation between ccBioZ and gold standard RIP with and without SQI processing.

Results obtained when performing the ccBioZ technology evaluation are shown in Figure 5. The SQI coverage results from Figure 5A, obtained by processing the previous data collection presented in [10], were compared against the results from Figure 5B, corresponding to the data in this collection. It is clear that the improvements applied to the ccBioZ system resulted in a significant increase of the data quality. Specifically, the median coverage increased from 7% to 58%. Consequently, the overnight coverage of ccBioZ-based respiratory activity was increased. Furthermore, the errors obtained in respiration rate were reduced when applying the SQI signal preprocessing, as shown in Figure 5C,D. The average correlation between ccBioZ signal and gold standard RIP from PSG also showed an important improvement when applying the SQI preprocessing (Figure 5E).

An overview of segments labeled as low quality with respect to OSA severity and sleep stages can be found in Table 2. Comparison of ccECG to ccBioZ showed that a substantially higher amount of ccECG epochs were removed by data quality assessment. For ccBioZ, 45% of obstructive apneas were removed and 34% of obstructive hypopneas. The possible reason is that an apnea is a complete cessation of breathing, which may show as a flat line in the breathing signal. A hypopnea still presents the normal wave pattern but with a reduced amplitude. This difference is less prominent in the cardiac signal. Furthermore, a large amount of wake epochs were discarded for both modalities. This is most probably due to movements during wake periods, which disturb signal acquisition.

### 4.2. Original Sleep–Wake Classification

The original ECG network from [5] was trained using CNN_Train and CNN_Val and applied onto the (cc)ECG recordings from dataset Test (see Figure 3A). A drop in performance was observed from full ECG recording (κ=0.18) to clean ECG (κ=0.08). This was most probably due to the large amount of discarded epochs (see Table 2). Evaluation of the ECG network with the clean ccECG resulted in a similar κ of 0.11. Therefore, it seemed that the ECG network generalized well for capacitively-coupled data. As the performance is, however, low, no strong conclusions can be drawn regarding generalization.

Similarly, the original RIP network was trained using CNN_Train and CNN_Val and was tested with RIP and ccBioZ recordings of dataset Test. Using all the segments of the RIP recordings resulted in a mean κ of 0.33, and the clean RIP data in a κ of 0.32. For the clean ccBioZ dataset, only a mean κ of 0.12 was reached. Thus, as opposed to the ECG network, the RIP network certainly did not generalize for ccBioZ recordings.

### 4.3. Augmented RIP Sleep–Wake Classification

Due to missing generalization of the RIP network, it was trained ten times in parallel, using min-max normalization and data augmentation (see Section 3.3). Then, the network with the highest κ score for CNN_Test was selected and this network achieved an average κ of 0.49. The performance of the selected network on all other datasets is listed in Table 3. The augmented RIP network reached a κ of 0.21 on the clean ccBioZ recordings, as opposed to a κ=0.12 with the original network. In addition, performance increased significantly to κ=0.23 (Wilcoxon signed rank test, *p* < 0.001) when including both high- and low-quality segments of the ccBioZ data. This improvement was most probably due to the doubled amount of wake epochs in the full recording compared to the clean set (see Table 2).

Furthermore, the RIP network was merged with the ECG network to form a multimodal network. As shown in Table 3, the augmented RIP network performed better than the multimodal network for datasets No, Mild, Mod, and Test. For the Test dataset, this was valid for both the PSG-based classifications and for those based on ccBioZ. These results were confirmed by the Wilcoxon signed rank test (*p* < 0.05).

**Figure 6:**
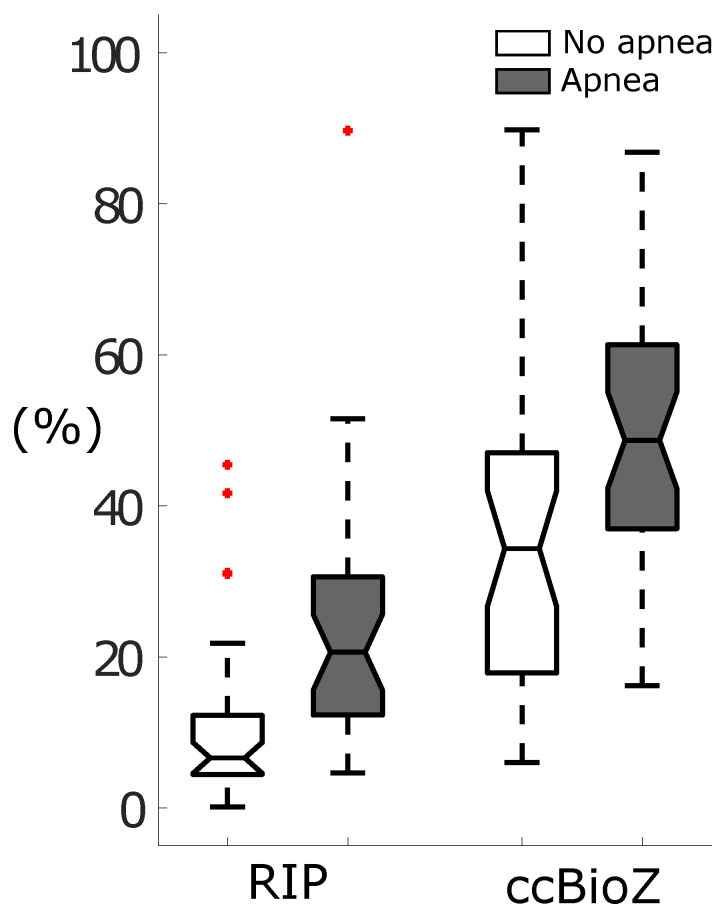
The number of false wake predictions per total amount of true wake epochs after sleep–wake classification on the full ccBioZ recordings. A distinction was made between epochs without (white) and with apneas (grey).

It was expected that apneas would increase the number of false positives, even more in the case of movement-sensitive acquisition systems. To investigate this, the number of false wake predictions per total amount of true wake epochs was calculated for each patient for both non-apneic as apneic segments. This was performed for the RIP signal as well as for the full ccBioZ recordings. The result is illustrated in Figure 6. The percentage of false wake predictions per patient without apneas was significantly larger for ccBioZ (Wilcoxon signed rank test, *p* < 0.001). For both modalities, this percentage increased when apneas occurred. Approximately 50% of apneic epochs recorded by ccBioZ resulted in a false wake prediction, as opposed to 20% using RIP.

### 4.4. Detection of OSA Patients

The confidence thresholds for sleep-predicted epochs, $T_{sleep}$, and wake-predicted epochs, $T_{wake}$, were based on the median and SD of p(sleep) and p(wake), respectively, of the sleep–wake prediction on CNN_Test. This resulted in $T_{sleep}=0.84-0.04=$ 0.80 and $T_{wake}=0.69-0.03=0.66$. For OSA patient detection, the percentage of uncertain sleep epochs, using $T_{sleep}$, was of importance as well as the percentage of sleep–wake transitions. Similarly as in [5], thresholds for these detection indices were defined by an ROC analysis based on the datasets No, Mild, Mod, and Sev. For “% Uncertain Sleep Epochs” in these datasets, a threshold of 66% yielded an Sp of 92% and Se of 35%. For “% Transitions”, a threshold of 24.3% yielded an Sp of 92% and Se of 39%.

Performance of the bivariate patient detection model on the clean ccBioZ, the full ccBioZ, and full PSG RIP recordings of the Test dataset can be found in Table 4. A distinction was made between detection of AHI ⩾ 15 and AHI ⩾ 30. For the three different subdatasets, the method achieved the highest κ for detection of severe OSA patients (AHI ⩾ 30). Using the full ccBioZ recording, a κ of 0.39 and accuracy of 69.4% was reached, compared to κ of 0.61 and accuracy of 80.6% for PSG RIP signals. For detection of AHI ⩾ 15, the specificity was 80% for both full ccBioZ and full PSG RIP.

**Figure 7:**
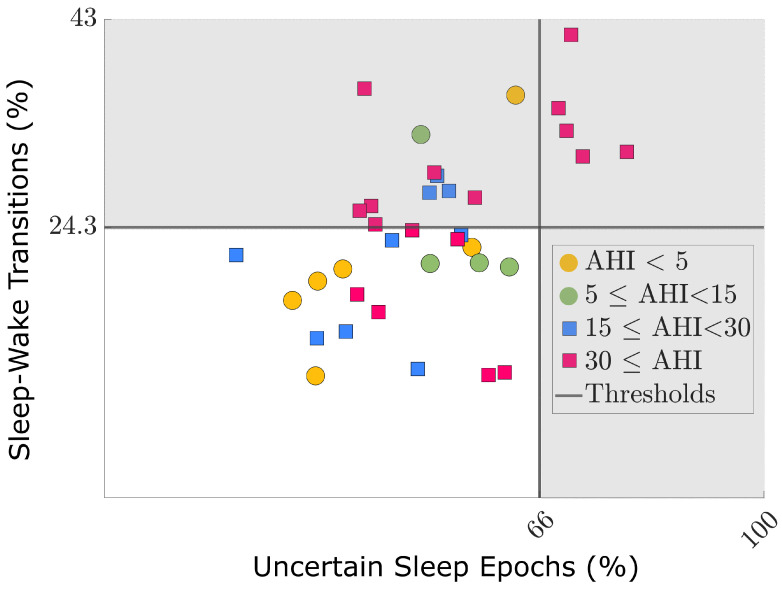
Application of the OSA patient detection model on the full ccBioZ recordings of the Test dataset. Circles represent AHI < 15 and squares AHI ⩾ 15, and the lines are the index-specific thresholds. The model identified a patient as being at risk of OSA (AHI ⩾ 15) if *at least one of both* metrics exceeded a selected threshold (grey area). For this, the model yielded a specificity of 80%. Five of the detected patients exceeded the thresholds of *both* detection indices (red squares in upper right quadrant), who were identified as being at risk of OSA with 100% accuracy. On the other hand, two patients were falsely detected as being at risk of OSA, indicated as a green and yellow dot in the upper left quadrant.

The outcome of the bivariate OSA risk-detection model using full ccBioZ recordings is depicted in Figure 7. In Section 3.4, it was defined that patients exceeding *at least one* of the two thresholds of the detection indices were identified by the model as being at risk of OSA (AHI ⩾ 15). In Figure 7, these patients are located in the grey area. Five of the detected patients exceeded the thresholds of *both* detection indices (red squares in upper right quadrant), who were identified as being at risk of OSA with 100% accuracy. This accuracy was reported in [5] as well. On the other hand, two patients were falsely detected as being at risk of OSA, indicated as a green and yellow dot in the upper left quadrant in Figure 7. These patients presented a relatively large percentage of sleep–wake transitions in the predicted hypnogram.

## 5. Discussion

### 5.1. Augmented CNN-Based Sleep–Wake Classification

This study applied a sleep–wake classifier that was proposed in [5] on ccBioZ data, which resulted in a performance drop compared to testing with PSG segments. Hence, it was suggested that the performance decrease was caused by the different dynamics of the ccBioZ signal compared to RIP. Therefore, the current study improved the sleep–wake classifier for ccBioZ analysis. This was achieved by changing the normalization scheme and applying data augmentation during training, based on PSG segments. This procedure aimed to decrease the undesired sensitivity of the network for amplitude differences as well as enhance the translation to the capacitive-coupled sensor and potentially other sensors.

The augmented RIP network improved enough to outperform the augmented multimodal network for datasets No, Mild, Mod, and Test. In addition, the performance on these datasets was similar or superior to the original multimodal network of [5]. The superior performance of the unimodal RIP network is an important asset as only a single modality is required. Moreover, the ccBioZ signal is less affected by environmental noise, as more control over the signal is possible due to its active measurement nature, as opposed to the passive measurement of ccECG.

The classifier augmentation step improved sleep–wake predictions using ccBioZ, but the performance did not reach the level of RIP-based predictions. The results in Figure 6 show more false positive wake predictions for ccBioZ compared to RIP, probably due to the increased sensitivity for movement [11]. The presence of apneas enforced this effect. It is noted, however, that apneas might result into micro-awakenings that are not clinically annotated, as discussed in [5]. These micro-awakenings can be related to movements, affecting the ccBioZ signal and leading to wake predictions. Thus, it can be assumed that a portion of “false” wake predictions can be associated with micro-awakenings.

Comparison of the classifier to the literature is difficult, on the one hand, as studies generally use a sequence of multiple epochs as input. Korkelainen et al. [17] and Dietz-Terjung et al. [18] developed deep learning networks for Wake-REM-NREM classification in suspected OSA patients, respectively, based on PSG PPG and PSG RIP with actigraphy. Both studies reached a *k* score larger than 0.60. For this, sequences of, respectively, 25 epochs and 100 epochs of 30 s were fed to the networks. As ccBioZ recordings are prone to motion artefacts due to the free-moving conditions [11], these algorithms seem less suitable for sleep staging of ccBioZ data. The network of this study predicts epoch-by-epoch and is, therefore, less dependent on long ranges of good quality data. On the other hand, few studies have performed sleep staging based on capacitively-coupled signals in OSA patients. In fact, to the best knowledge of the authors, no studies were found based on bioimpedance. Lee et al. did investigate ccECG for REM sleep and wakefulness [7]. However, only qualitative results on a single patient were reported.

### 5.2. Detection of OSA Patients

The thresholds for OSA patient detection indices from [5] were updated using the unimodal augmented RIP network. The threshold for “% Uncertain Sleep Epochs” changed from 64% to 66% and for “% Transitions” from 24% to 24.3%. Thus, these thresholds only changed minimally, although a unimodal instead of a multimodal network was used. This again showed that most information was contained in the respiratory signal when predicting sleep–wake stages from single 30 s epochs. Furthermore, the OSA patient detection model was applied on an independent test set, recorded with the novel modality, ccBioZ.

It was observed that the detection of OSA patients using the full ccBioZ recording was superior to the one obtained using only the clean subset (see Table 4). The most probable reason is that cleaning the dataset involved removing on average 34% of the obstructive hypopneas, 45% of the obstructive apneas, and 51% of wake epochs. Wake epochs are, however, potential OSA indicators as these contribute to the percentage of sleep–wake transitions. Similarly for apneas, it was shown that these caused more wake predictions, resulting into more sleep–wake transitions. Thus, the presence of substantially more (false) wake predictions in the full ccBioZ recordings was applied as useful information for high-risk OSA patient detection, and cleaning of the dataset meant removing essential information. The study by Deviaene et al. on apnea detection from capacitively-coupled data presented similar findings [9]. They reported a decreased sensitivity when only including the high-quality segments of the capacitive data, since a large portion of apneas was removed due to data cleaning. For future studies, a relation between these wake predictions and periodic limb movements should be investigated as well. People with this disorder experience repetitive, uncontrolled movement of their lower limbs during sleep, and it is strongly associated with OSA [19].

Currently, clinicians make use of standardized OSA screening questionnaires, which can be regarded as an *unobtrusive* tool. Chiu et al. [20] compared the screening performance of commonly used questionnaires and found the STOP-BANG questionnaire (SBQ) to be a superior tool for detecting mild, moderate, and severe OSA patients. However, the Se of SBQ is high (AHI ⩾ 15: 90%, AHI ⩾ 30: 93%) at the expense of low Sp (AHI ⩾ 15: 36%, AHI ⩾ 30: 35%). In comparison, this study aimed for higher Sp values (see Section 3.4). This design goal was fulfilled as Sp values of 80.0% (AHI ⩾ 15) and 73.7% (AHI ⩾ 30) were reached using ccBioZ, and 80.0% (AHI ⩾ 15) and 84.2% (AHI ⩾ 30) using PSG RIP. The OSA detection based on ccBioZ could be used additionally to SBQ, as the Sp values of both are complementary, and both methods are fully unobtrusive.

The patients detected by the current model (16 patients in grey area in Figure 7) most probably have stronger physiological dynamics compared to the negative patients (21 patients in white area in Figure 7). These strong dynamics complicate a correct sleep–wake prediction. Indeed, when ignoring the 16 patients identified by the model as being at risk of OSA, the performance of sleep–wake classification based on ccBioZ significantly increased from κ=0.23 to κ=0.28. Thus, the hypnogram of these detected patients was less accurate, but this was made evident by the fact that these patients would be prioritized for a refined clinical diagnosis. On the other hand, two patients with an AHI < 15 were falsely detected as being at risk. Excessive movements or a suboptimal sensor coupling could have caused this. To resolve this doubt, a multiple night follow-up is advised to mitigate any instabilities in sleep behavior or recording quality.

### 5.3. Data Quality Assessment and Technology Evaluation

A drop in the ccECG data coverage with respect to a previous study ([10]) was seen. The cause of this decrease was a deterioration of the conductive textile used in the prototype from Figure 1. Oxidation of the sensors was likely to be the source of degradation. This is expected to be solved when using materials less prone to oxidation or using conductive textile electrodes with a coating or sealed in materials such as polyurethane. On the other hand, a significant increase in ccBioZ signal coverage was identified as a result of improvements in the acquisition system. A comparison of both capacitively-coupled signals against the PSG gold standard did confirm that the application of SQI-based preprocessing results in a significant decrease in the error of extracted cardiac and respiratory features. This means high-quality segments can be identified automatically, despite the fact that ccECG and ccBioZ signals are affected by motion artifacts.

As OSA is associated with restlessness and frequent movement, the capacitively-coupled sensors are more affected by motion artifacts in OSA patients. This results in lower coverage than in subjects without OSA, where coverage metrics of up to 72% have been reported [6]. Therefore, the capacitively-coupled sensors cannot serve as a complete replacement for PSG, but rather as an early-stage unobtrusive monitoring tool. On the other hand, this characteristic of the ccBioZ (i.e., susceptibility to motion artifacts) was exploited for identification of OSA patients. Moreover, the sensor could be suited for dedicated monitoring in elderly care centers [21,22]. In this setting, unobtrusive sensor systems are required to enable continuous monitoring. The sensor mattress itself can be installed quickly and does not contain electrodes which require trained personnel to attach to the patient. The proposed framework could monitor the cardiac and respiratory status of patients and detect those requiring more urgent care. Alternatively, the mattress and proposed methods could also serve for follow-up of CPAP treatment to check for improved health conditions. The status of the patient can then be monitored by following the path of their index values in the bivariate model as presented in Figure 7.

## 6. Conclusions

Suspected OSA patients would strongly benefit from comfortable home diagnosis. Within this context, the potential of capacitively-coupled cardiac and respiratory sensors integrated in a mattress was assessed for sleep monitoring in suspected OSA patients. A significant increase in ccBioZ signal coverage was observed as a result of improvements in the acquisition system. Moreover, SQI-based preprocessing correctly identified high-quality segments. On top of this evaluation, this is the first study to develop a sleep–wake classifier for suspected OSA patients using the ccBioZ signal. This inevitably resulted in false wake predictions by the high sensitivity of the sensor for motion. Nonetheless, these false wake predictions were applied as information for OSA patient detection to prioritize these patients with high specificity for a hospital diagnosis. Overall, the algorithmic framework in combination with the characteristics of the mattress could be suitable for multi-night recordings as well as applications in long-term elderly care.

## Figures and Tables

**Table 1:** Demographic and clinical information of the study datasets. N is the number of subjects in the dataset, with the number of non-apneic patients indicated between brackets. The column AHI indicates the average and SD of the AHI over all patients in the corresponding dataset. The last colum Wake indicates the time spent in wakefulness, averaged over all patients in the corresponding dataset. Within brackets, the percentage of wakefulness in the total recording is given. All datasets contained PSG recordings. The unseen dataset Test additionally contained ccECG and ccBioZ recordings.

	N	Age (Years)	BMI (kg/m^2^)	Male	AHI (1/h)	Wake (h)
	(AHI < 5)	Mean (SD)	Mean (SD)	%	Mean (SD)	Mean (%)
CNN_Train	39 (13)	41.3 (11.1)	29.0 (6.3)	54	5.5 (2.4)	1.55 (18)
CNN_Val	17 (9)	42.4 (14.0)	32.3 (8.0)	53	6.0 (2.2)	1.78 (20)
CNN_Test	26 (13)	38.8 (10.9)	27.5 (6.7)	50	4.8 (2.6)	1.40 (17)
No	13	39.0 (11.2)	25.6 (5.3)	23	2.2 (1.3)	1.23 (14)
Mild	24	43.3 (13.8)	28.7 (6.4)	58	8.7 (2.9)	1.96 (23)
Mod	19	54.1 (10.7)	31.5 (4.4)	63	20.4 (4.3)	1.99 (24)
Sev	35	54.8 (12.4)	32.3 (4.3)	74	61.9 (20.1)	1.97 (24)
Test	36 (6)	50.8 (10.6)	30.6 (5.4)	78	38.6 (29.6)	2.34 (27)

AHI: Apnea–Hypopnea Index. No: AHI < 5, Mild: 5 ⩽ AHI < 15, Mod: 15 ⩽ AHI < 30, Sev: AHI ⩾ 30. SD: standard deviation.

**Table 2:** Overview of low-quality (LQ) labeled segments for ccECG and ccBioZ with respect to occurrence of apneas and sleep stages.

	ccECG LQ (%)	ccBioZ LQ (%)
NoApn	67 ± 29	39 ± 18
Hobs	67 ± 30	34 ± 20
Aobs	64 ± 42	45 ± 35
Wake	75 ± 30	51 ± 18
Sleep	64 ± 30	33 ± 20

Hobs: Obstructive Hypopnea. Aobs: Obstructive Apnea.

**Table 3:** Mean Cohen’s Kappa scores of sleep–wake classification using the unimodal (ECG, Augmented RIP) and multimodal (ECG with Augmented RIP) networks. These networks were trained on PSG recordings of patients with AHI < 10 and tested on patient data with varying AHI, as well as on unobtrusive ccECG and ccBioZ data. The main results are indicated in grey, and the best results are given in boldface.

	ECG	Aug RIP	ECG + Aug RIP
CNN_Train	0.27	0.49	0.51
CNN_Val	0.27	0.32	0.31
CNN_Test	0.26	0.49	0.45
No	0.20	**0.48**	0.43
Mild	0.24	**0.47**	0.44
Mod	0.24	**0.43**	0.41
Sev	0.17	0.26	0.29
Test			
$\mathrm{ECG}{\;}^{a},\mathrm{RIP}{\;}^{a}$	0.18	**0.39**	0.31
$\mathrm{ECG}{\;}^{b}$	0.08	-	-
$\mathrm{ccECG}{\;}^{b}$	0.11	-	-
$\mathrm{ECG}{\;}^{c},\mathrm{RIP}{\;}^{c}$	0.10	0.29	0.26
$\mathrm{ECG}{\;}^{c},\mathrm{ccBioZ}{\;}^{c}$	0.10	0.21	0.16
${\mathrm{ECG}}^{a},\mathrm{ccBioZ}{\;}^{a}$	0.18	**0.23**	0.17

Aug: Augmented CNN for respiratory input. ${}^{a}$ Including all segments. ${}^{b}$ Only segments labeled as high-quality ccECG. ${}^{c}$ Only segments labeled as high-quality ccBioZ.

**Table 4:** Performance of the OSA patient detection model on the high-quality ccBioZ, full ccBioZ and full PSG RIP recordings of the Test dataset. The main results are indicated in grey, of which the best results are in bold.

	ccBioZ HQ		ccBioZ Full		PSG RIP Full	
	AHI	AHI	AHI	AHI	AHI	AHI
	⩾ 15	⩾ 30	⩾ 15	⩾ 30	⩾ 15	⩾ 30
κ	0.22	0.23	0.26	0.39	0.26	0.61
Acc (%)	63.9	61.1	61.1	69.4	61.1	80.6
Se (%)	65.4	70.6	53.9	64.7	53.9	76.5
Sp (%)	60.0	52.6	**80.0**	73.7	80.0	**84.2**
DOR	2.84	2.66	4.68	5.14	4.68	17.3

HQ: High-quality segments. Acc.: Accuracy. Se: Sensitivity. Sp: Specificity. DOR: Diagnostic odds ratio.

## Data Availability

The datasets presented in this article are not readily available because they contain information that could compromise the privacy of research participants and are subject to the European data-privacy policy regulations. The authors will try to provide an anonymized version of the dataset in compliance with the privacy policy of the University Hospitals of Leuven, which is the owner of the data. Requests to access the datasets should be directed to Carolina Varon, carolina.varon@esat.kuleuven.be.

## Abbreviations

The following abbreviations are used in this manuscript:

Acc	Accuracy
AHI	Apnea–hypopnea index
Aobs	Obstructive apnea
BPM	Beats per minute
ccBioZ	Capacitively-coupled bioimpedance
ccECG	Capacitively-coupled electrocardiography
CNN	Convolutional neural network
DOR	Diagnostic odds ratio
Hobs	Obstructive hypopnea
OSA	Obstructive sleep apnea
PSG	Polysomnography
REM	Rapid eye movement (sleep)
RIP	Respiratory inductance plethysmography
SD	Standard deviation
Se	Sensitivity
Sp	Specificity
